# Manufacturing and performance evaluation of medical radiation shielding fiber with plasma thermal spray coating technology

**DOI:** 10.1038/s41598-021-01897-w

**Published:** 2021-11-17

**Authors:** Seon-Chil Kim, Jun-Sik Son

**Affiliations:** 1 Department of Biomedical Engineering, Keimyung University School of Medicine, Daegu, Korea; 2 Korea Textile Development Institute, Daegu, Korea

**Keywords:** Biomedical engineering, Radiography

## Abstract

Lead, which has been used for radiation shielding in medicine, is currently sought to be replaced by an eco-friendly shielding material. Therefore, it should be replaced with shielding materials possessing excellent processability and radiation shielding performance similar to that of lead. In this study, a new process technology was developed focusing on the processability of tungsten, a representative eco-friendly shielding material. It is difficult to reproduce the shielding performance when using the method of coating nonwoven fabrics with a liquid using tungsten powder on a polymer material, which is adopted to ensure the flexibility of the shielding fabric. To address this, tungsten powder was sprayed on the fabric using a plasma thermal spray coating process and coated to a thickness of 0.2 mm to evaluate the shielding performance. Compared to standard lead with a thickness of 0.2 mm, the shielding efficiency differed by approximately 15%. Since the developed process can maintain the amount of injection in an area, it is possible to ensure the reproducibility of the shielding performance and automated process for mass production. This approach is economically feasible as it does not entail the mixing of polymer materials; hence, it can be used for preparing radiation shielding clothing for medical institutions.

## Introduction

Lead is mainly used for the manufacturing of radiation shields in medical institutions. Lead features excellent X-ray shielding performance, and it is widely used in various forms in medical institutions owing to its excellent processability and economic efficiency^1,2^. However, the increased use of lead poses a risk of lead poisoning, owing to the greater exposure of medical staff and patients to lead; furthermore, the disposal of lead is also associate with certain environmental concerns^3^. To address this problem, materials composed of a mixture of various eco-friendly shielding materials, such as tungsten, bismuth, barium sulfate, and antimony, and inorganic materials have recently been employed as radiation shields^4,5^.

Medical institutions generally adopt shielding suits, which facilitate user activities owing to their use of lightweight materials and flexibility^6^. The shielding fabric in a shielding suit should possess sufficient flexibility such that it does not interfere with the activities of the medical personnel; however, the flexibility of the shielding suit has a direct relationship with the shielding performance^7^. For instance, to improve flexibility, the content of the polymer material used during shield manufacturing needs to be increased; however, this would reduce the density of the particle structure within the shielding material, resulting in a degradation in the shielding performance^8^. Conversely, if the content of the polymer material is decreased, the bonding between the shielding materials is reduced, and cracks may appear when using a thin shielding sheet^9^. Therefore, the shielding fabric used in medical institutions must possess certain thickness, flexibility, and tensile strength and exhibit complex conditions that enable a shielding performance similar to that of lead^10,11^. Therefore, the ideal method is to process and use the shielding material alone; however, it is difficult to manufacture the material in the desired shape owing to limitations in processability depending on the material^12^.

Radiation shielding clothing currently used in medical institutions is mainly manufactured by mixing lead powder and polymer materials such as rubber; the shielding performance of most of these suits depends on the amount of lead powder content^13,14^. When a shielding sheet composed of a polymer material and a shielding material mix is mass-produced, various variables such as process technology, mixing conditions, and mixing amount of the shielding material are involved in the production process. Accordingly, shielding sheets are produced via small quantity orders^15^. Therefore, it is considerably important to study the reproducibility of the shielding performance according to the process technology used for the shielding fabric. The shielding performance cannot be determined simply based on the amount of shielding material added^16^. Many factors such as density and porosity affect the shielding performance^17^. Consequently, to manufacture a suitable shield, a new manufacturing process technology that can satisfy the aforementioned various conditions needs to be developed.

When mass-producing radiation shields, the technology that injects the same amount of shielding material into the same area determines the reproducibility of shielding performance. However, it is difficult to obtain a direct proportional relationship between the shielding performance and the shielding material, owing to the various additional conditions, such as the base materials and additives employed in the process technology. Therefore, this study intends to present a novel plasma thermal spray coating process technology that can directly coat the metal particles of the shielding material; this approach achieves the same amount of shielding material over the same area and maintains the reproducibility of shielding performance.

In this study, tungsten was selected as an eco-friendly shielding material. Recently, many studies have focused on medical radiation shielding materials; it has been reported that the shielding performance of tungsten is similar to that of lead^18^. However, as tungsten is expensive and its processability is poor, tungsten powder and a polymer material are mixed and processed in the form of a sheet or film^19,20^.

The purpose of this study is to develop a shielding fiber that maintains flexibility through the process technology of coating tungsten powder directly on the fabric. A plasma spraying device was developed to coat tungsten on fibers through plasma thermal spray method, and aramid fibers resistant to high temperatures were woven with high density. The amount of tungsten must be uniformly dispersed for use in the mass production process. The shielding performance was controlled by varying the amount of tungsten powder and was compared with the direct shielding performance of lead. Therefore, this study proposes a new process technology for manufacturing shielding fibers that can control the shielding performance by adjusting the thickness of the coating on the shielding fabric through plasma thermal spray without mixing tungsten powder with a polymer.

## Methods

Tungsten, which was selected as the radiation shielding material, has an atomic number of 74, similar to the atomic number of lead (82), and a density of 19.25 g/cm^3^, which is considered a high density; it is also known to possess a shielding performance similar to that of lead^21^. The plasma thermal spray coating method was evaluated in this experiment to ensure that the prepared fabric is lightweight and flexible, such that it can serve as a replacement for lead. In plasma thermal spray coating technology, tungsten particles are sprayed at high temperatures of up to 1700 °C.

**Figure 1:**
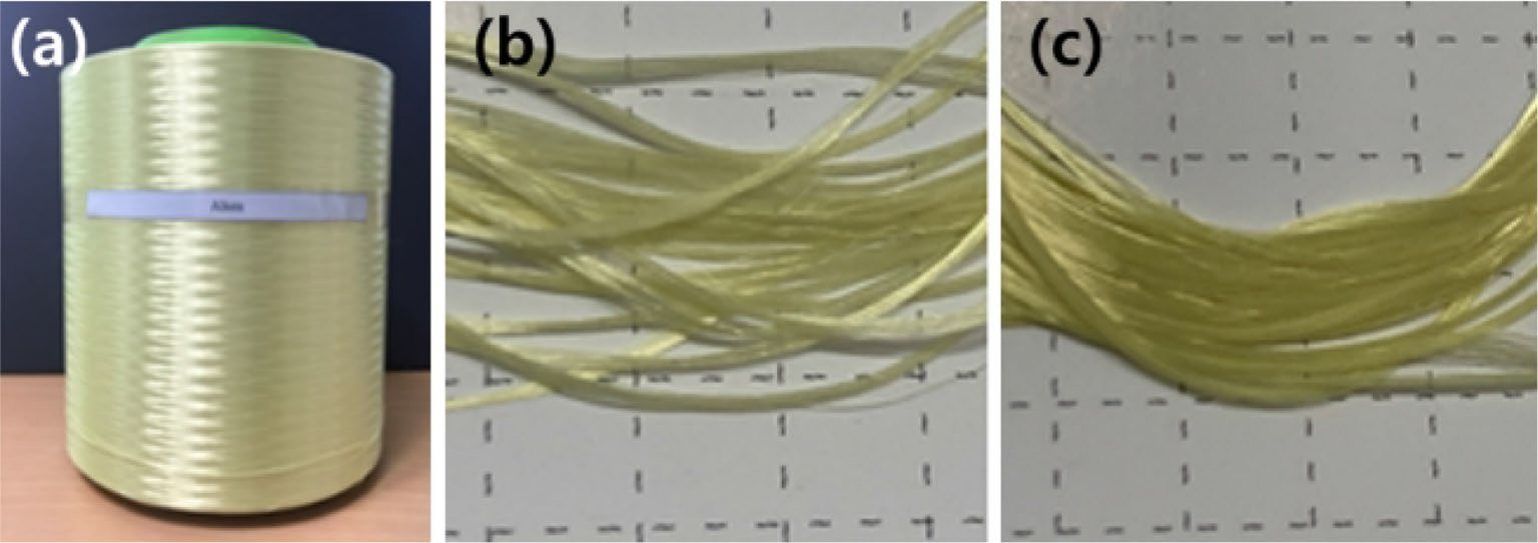
Para-aramid double-weave yarn: **(a)** para-aramid yarn (ALKEX), **(b)** 200 denier para-aramid yarn (surface), and **(c)** 400 denier para-aramid yarn (rear).

**Figure 2:**
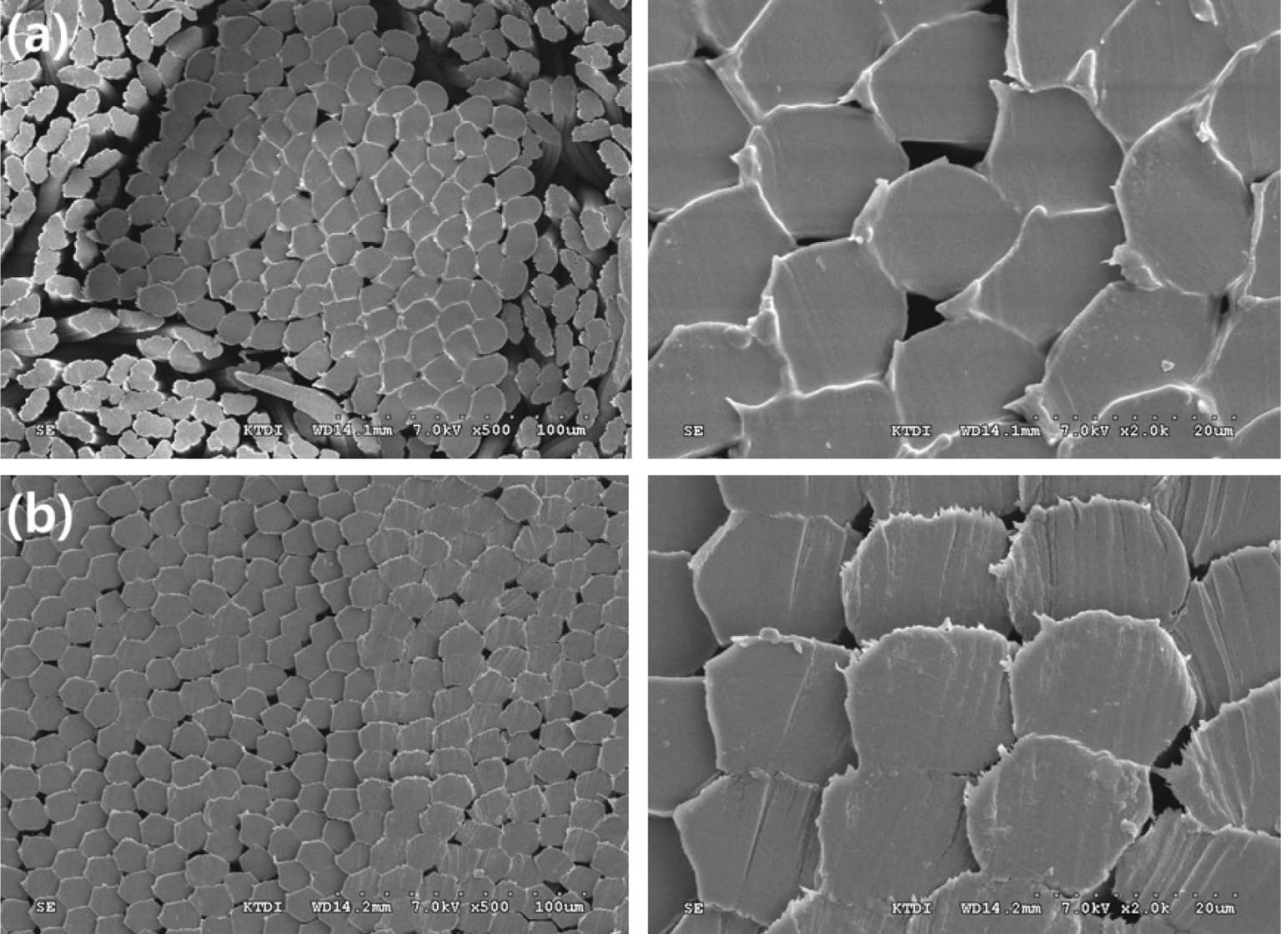
SEM images of para-aramid yarn: **(a)** 500 × and 2000 × magnification images of 200 denier para-aramid yarn used on the fabric surface and **(b)** 500 × and 2000 × magnification images of 400 denier para-aramid yarn used on the rear of the fabric.

The conditions used in this technique ensure adhesion to the fabric after spraying the tungsten particles at high temperatures and that the fabric can withstand high temperatures. First, the fabric was weaved using an aramid double-weave fabric. To weave the aramid double-weave fabric, para-aramid yarn (ALKEX), featuring high strength and high heat resistance, was used, as shown in Fig. 1. A 200-denier yarn was applied to the surface, whereas a 400-denier yarn was applied to the rear. Thus, a para-aramid double-weave fabric that can be fixed without undergoing deformation at high temperatures was realized. The scanning electron microscopy (SEM) images of the cross-section of the para-aramid yarn used are depicted in Fig. 2.

During this process of spraying tungsten on a para-aramid fabric with high heat resistance, a thicker yarn was employed at the rear of the fabric to reduce the air gap caused by the fabric structure. Therefore, the surface and the rear sides were composed of para-aramid yarns with different fineness. For high-density double-weaving, the fabric structure was designed as shown in Table 1.

**Table 1:** Design table for aramid double-weave organization.

Division		Warp	Weft	Warp density	Weft density	Weaving width
Aramid double-weave fabric	Surface layer	Aramid 200 denier	Aramid 300 denier	1200	100 T	1 m
	Back layer	Aramid 400 denier	Aramid 400 denier	800		
	Organizational chart					

**Figure 3:**
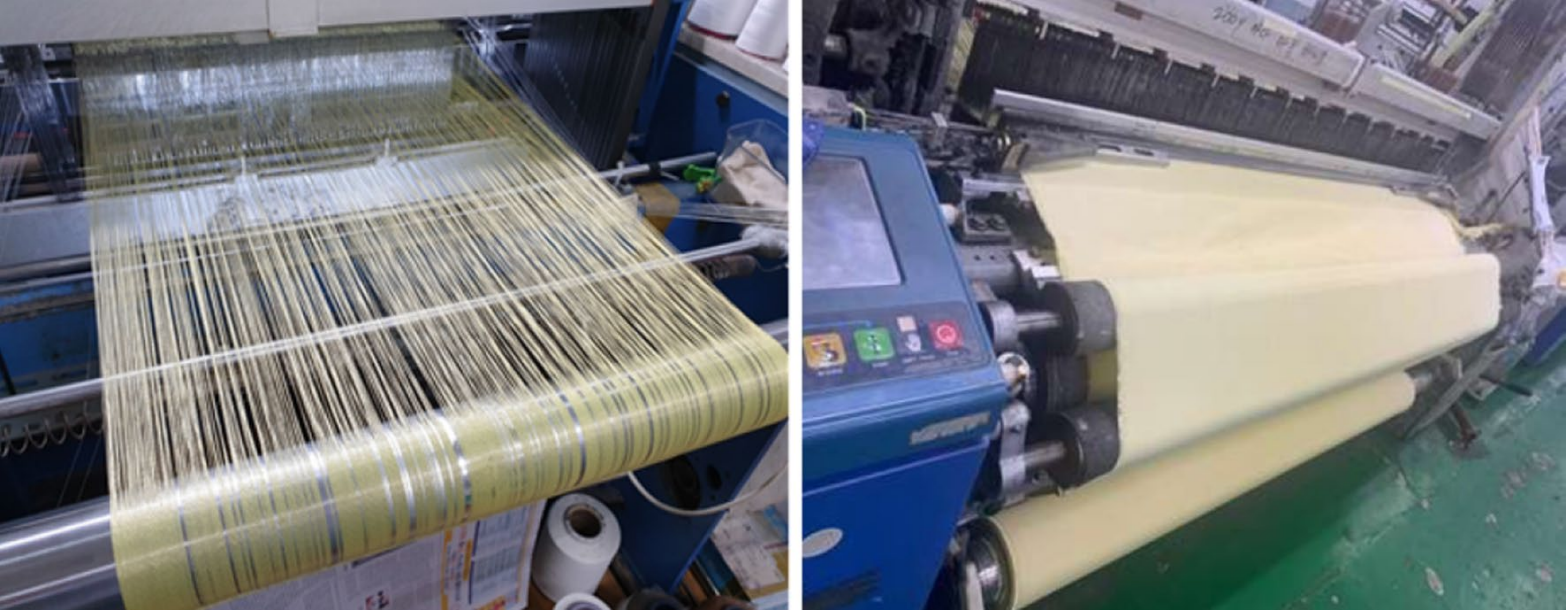
Para-aramid double-weave fabric weaving process.

The fabric was weaved with the selected yarn using an air jet loom (Toyota. Co., Model: Toyota 610), as shown in Fig. 3.

**Figure 4:**
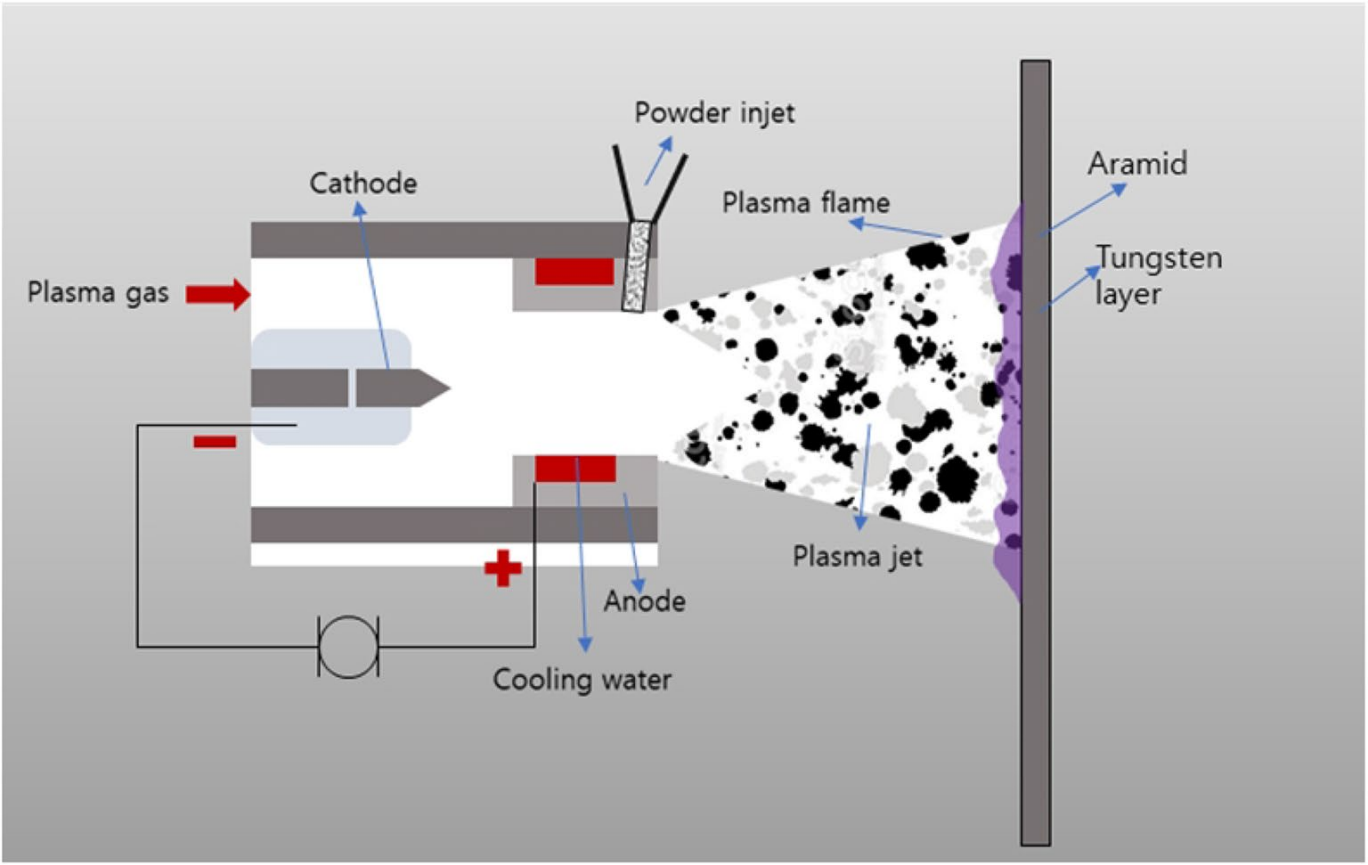
Schematic of plasma thermal spraying method.

In this study, the plasma thermal spray coating method was employed, instead of the existing polymer binder coating method, to manufacture shielding clothing that can be used in medical institutions. Plasma thermal spray coating is a process wherein a thermoelectrically ionized gas partially melts a small amount of cobalt, an inorganic particle, and accelerates it to a high speed in a semi-molten state to thinly coat the surface of a fabric. This plasma thermal spray coating method is depicted in Fig. 4.

**Figure 5:**
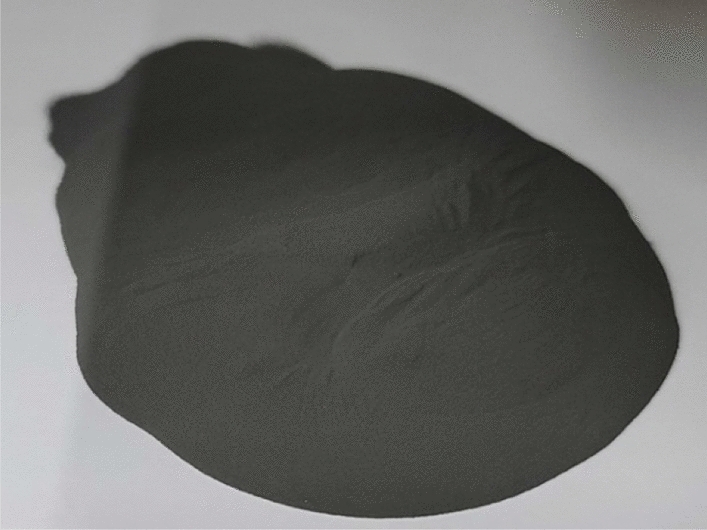
Tungsten powder.

The tungsten particles used as the shielding material were in the form of a powder, as shown in Fig. 5; the particles used were smaller than 500 nm. Tungsten has a melting point of 3422 °C, which is the highest among the elements, thus making it difficult to process it into the desired shape^22^. However, the plasma thermal spray coating method developed in this study does not melt the existing tungsten particles. Given that the tungsten powder is mixed with a small number of inorganic particles, a semi-molten state is achieved, and effective spraying is realized.

**Figure 6:**
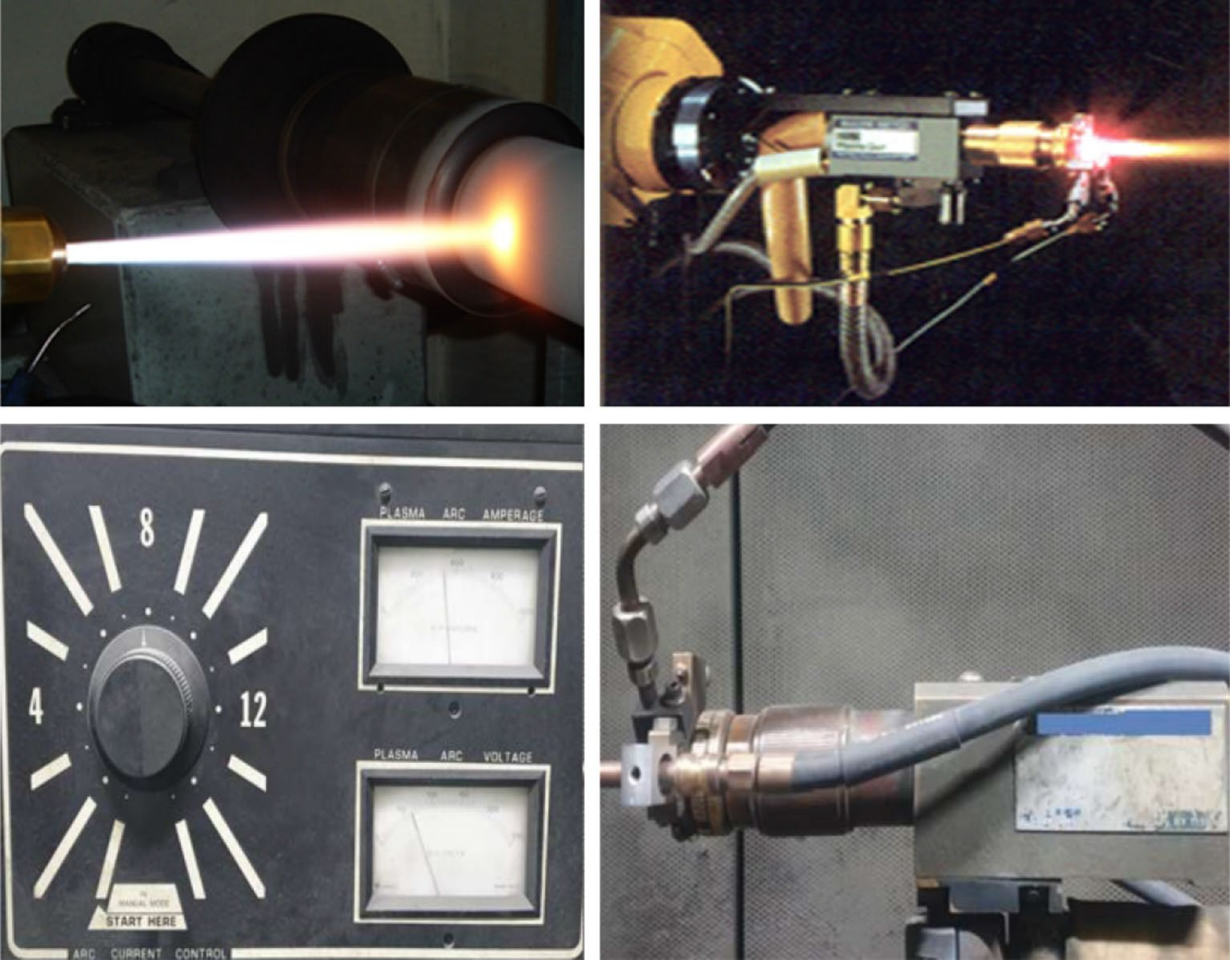
Equipment and control devices used in plasma thermal spraying process.

The process control parameters of the plasma thermal spraying method include the plasma output and spraying temperature, which affect the properties of the resultant tungsten coating. Figure 6 shows the equipment and control device used in the plasma thermal spraying process. The experiment was conducted under the conditions of a plasma output of approximately 80 kW and a spraying temperature of approximately 1,700 ℃. To ensure the same amount of material in the same area, the plasma movement speed was set to 5 mm/s based on the coating thickness of 0.2 mm, and tungsten particles were sprayed. Thus, a tungsten shielding fabric of 1 m × 1 m × 1.2 ± 0.02 mm was manufactured.

In radiation shielding, the number of metal atoms in a shielding material and the collision probability increase with an increase in the surface area per unit mass, thereby effectively attenuating the incident radiation and achieving a good shielding effect^23,24^. Therefore, assuming that the intensity of the incident radiation is ${I}_{0}$ and the linear attenuation coefficient of the shielding material is $\mu$, the transmittance intensity ($I$) when the radiation passes through the fabricated shielding fabric of thickness ($t$) is given as follows^25^:

1 $$\begin{array}{c}I={I}_{0}{e}^{-\mu t} \#\end{array}$$

Although the areal density of the fabric varies depending on the yarn, it is possible to determine the mass attenuation coefficient to improve the damping ability by coating tungsten on the fabric using Eq. (2) ^26^.

2 $$\begin{array}{c}\frac{\mu }{\rho }=\frac{s}{m} In\left(\frac{I}{{I}_{0}}\right)\#\end{array}$$

where $s$ is the area in which the radiation attenuation effect can be expected and $m$ is the mass per unit area. For tungsten in the solid state, the density is 19.25 g/cm^3^; consequently, the coating and shielding effects occur through even dispersion of the shielding material particles.

**Figure 7:**
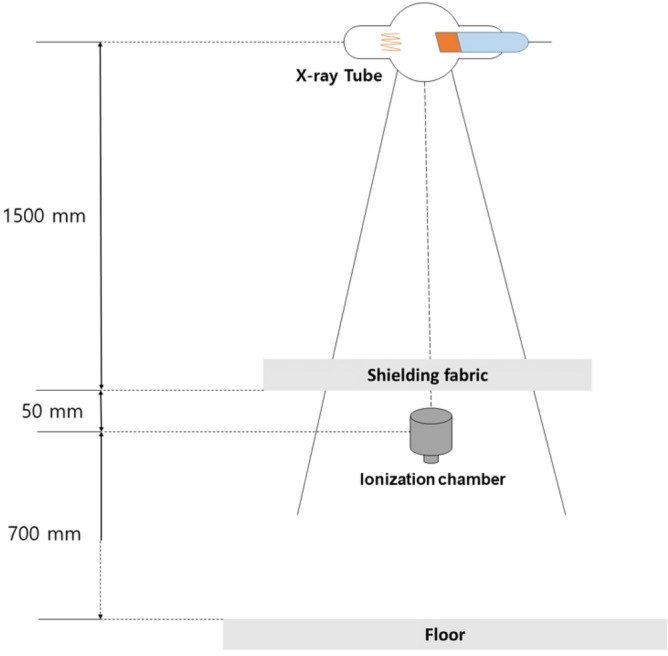
Shielding performance test schematic.

An optical microscope (OM, Axiotech 100 HD, Zeizz) and SEM (JSM-5410, Jeol) were employed to examine the cross-section and surface structure of the fabricated shielding fabric. The shielding performance evaluation of the fabric was performed as shown in Fig. 7 by applying the lead equivalent test method (KS A 4025: 2017)^27^. In addition, the shielding rate measurement was performed using Eq. (3) ^28,29^.

3 $$\begin{array}{c}S=\left(1-\frac{T}{{T}_{0}}\right)\times 100\#\end{array}$$

where $S$ is the shielding rate, ${T}_{0}$ is the incident dose (mR), and $T$ is the transmitted dose (mR). Specifically, $T$ is the exposure dose measured in the presence of the shielding fabric between the X-rays and the detector, and ${T}_{0}$ is the exposure dose measured without the shielding fabric between the X-rays and the detector. Under the measured dose conditions, the tube current was 200 mA and the irradiation time was 0.1 s. The exposure dose was measured 10 times using an X-ray generator (DK-525, Toshiba E7239X, Tokyo) and a calibrated ion chamber (Model PM-30, PR-18), and then the average value was used. In addition, the shielding performance was compared with the fabric developed using 0.2 mm standard lead (purity 99.8%).

## Results

**Figure 8:**
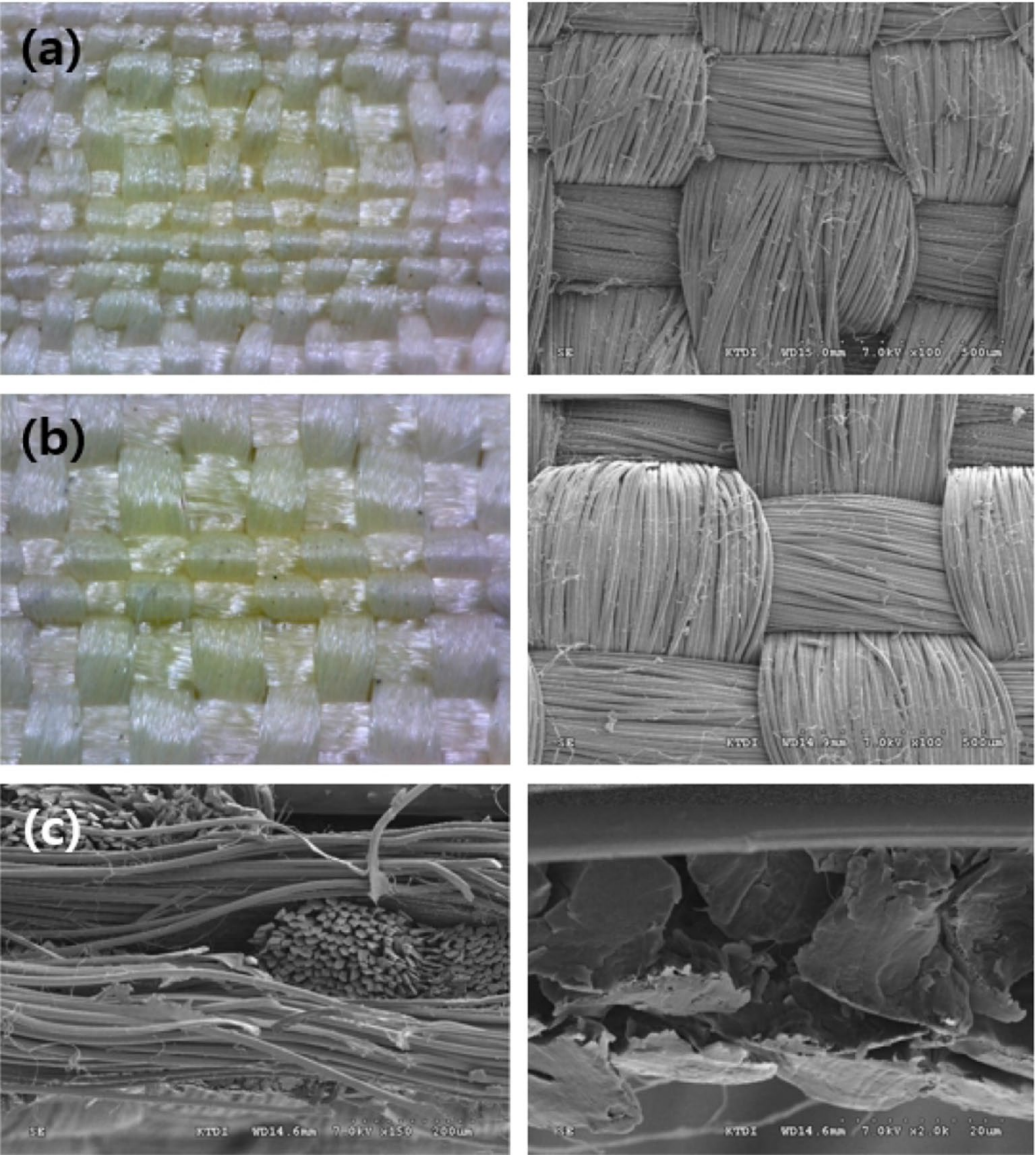
Para-aramid fabric. **(a)** 100 × magnified image of the surface of the fabric, **(b)** 100 × magnified image of the rear side of the fabric, **(c)** 150 and 2000 × magnified image of the cross section of the fabric.

Table 2 shows the characteristics of the double-weave fabric woven with a para-aramid yarn. Notably, the fabric exhibited high heat resistance, flexibility, and strength. Further, the tensile strength, tensile elongation, and tearing strength of the shielding fabric were high. The woven fabric was observed using an electron microscope as shown in Fig. 8, and it was confirmed that the surface of the fabric was generally rough before tungsten coating. In addition, the thickness of the fabric was measured to be 1.0 ± 0.04 mm. The woven fabric should have good adhesion between tungsten particles and the fabric with zero loss, even at high temperatures, and voids should be minimized. Fig. 8a shows the surface image depicting that the fabric has density, and (b) shows that the fabric could withstand high temperature and is slightly thicker to prevent voids. High-density weaving can also be confirmed from the cross-sectional image (c).

**Table 2:** Features of double-weave aramid fabric.

Tensile strength (KS K 0520:2015)		Tensile elongation (KS K 0520:2015)		Tearing strength (ISO 13937-2:2000)		Weight
Warp	3400 N	Warp	30.4%	Warp	330.27 N	3.24 g/10 cm^2^ (324 gsm ± 3%)
Weft	6000 N	Weft	7.8%	Weft	602.78 N

**Figure 9:**
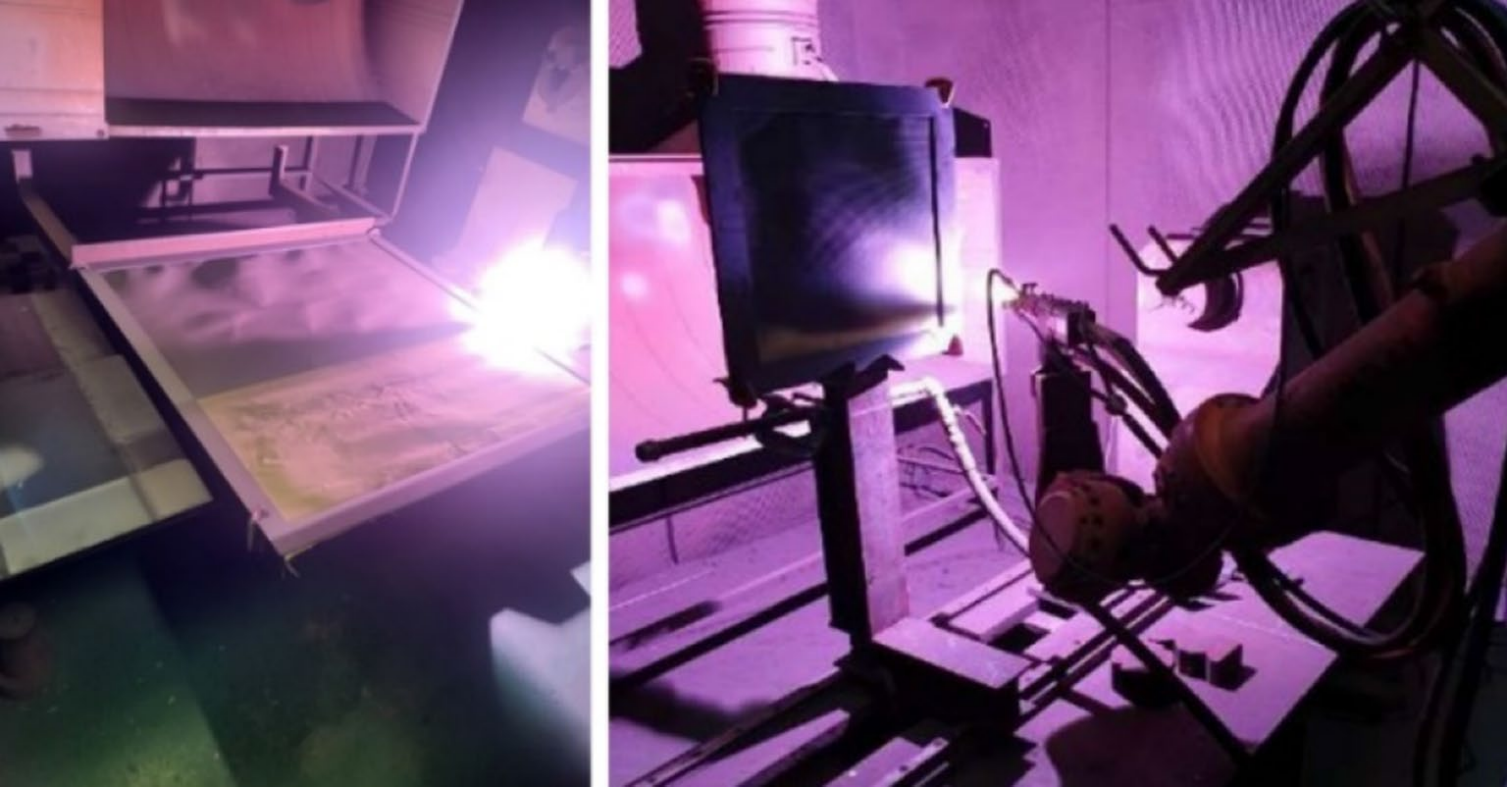
Plasma thermal coating–based tungsten spraying process.

The plasma thermal spray coating process was performed, as shown in Fig. 9, on the para-aramid fabric developed in this study. The distance between the shielding fabric and the spraying device was 100 mm, and powder injection was performed at a moving speed of 5 mm/s. Therefore, the devices used in the experiment were manufactured to enable such movement.

Table 3 lists the characteristics of the finished tungsten-coated shielding fabric. The overall strength of the shielding fabric was lower than that before the thermal spray coating, which can be explained by the weakening of the yarn owing to the high temperature. However, it can be seen that the thin film coating achieved due to the high temperature was successful.

**Table 3:** Features of para-aramid shielding fabric with tungsten coating.

Tensile strength (KS K 0520:2015)		Tensile elongation (KS K 0520:2015)		Tearing strength (ISO 13937-2:2000)		Weight
Warp	2800 N	Warp	27.9%	Warp	159.87 N	9.38 g/10 cm^2^ (938 gsm ± 3%)
Weft	3200 N	Weft	4.8%	Weft	305.77 N	

**Figure 10:**
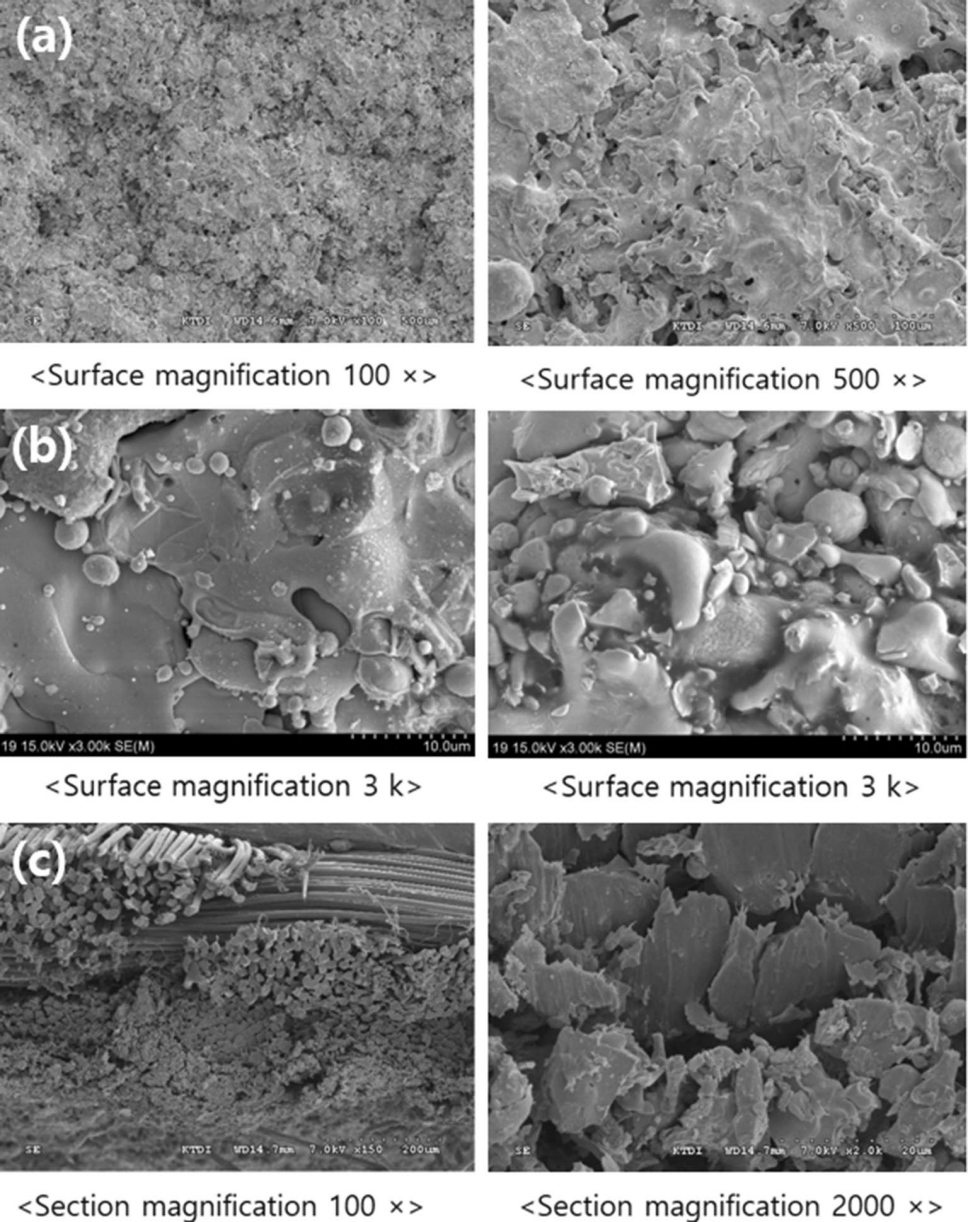
Tungsten-coated shielding fabric. **(a)** An enlarged image of the surface of the fabric, and **(b)** the distribution of tungsten particles in the enlarged surface image, **(c)** the enlarged cross-sectional image of the fabric.

**Figure 11:**
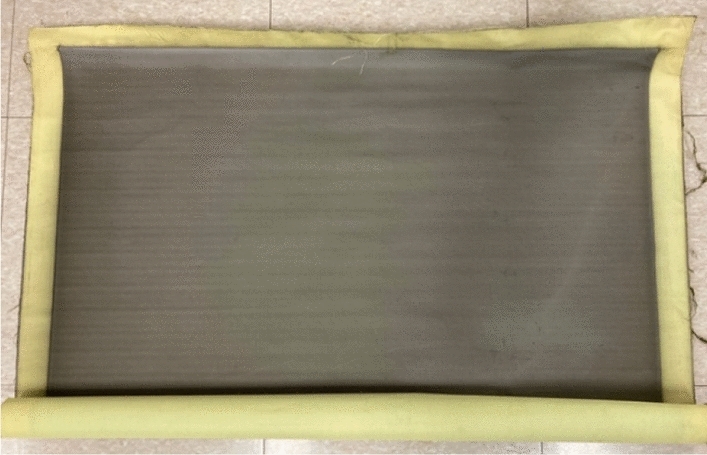
Finished tungsten-coated shielding fabric.

The woven tungsten-coated shielding fabric was observed using an electron microscope, as shown in Fig. 10, and its appearance is shown in Fig. 11. The tungsten particles are well fixed on the surface of the fabric after the plasma thermal, as shown in enlarged image of the surface of Fig. 10a,b. In particular, in Fig. 10b, tungsten particles coated on aramid fibers can be seen. Thus, it was confirmed that there were no problems such as resin agglomeration that occurs in the conventional method of coating the nonwoven fabric in a liquid form. Moreover, uniform dispersion was achieved. As shown in the surface enlarged image, the characteristics of the fabric were well expressed. The cross-sectional image analysis shows that the double weave fabric was well presented, and it can be confirmed that the tungsten is well settled without any loss such as yarn breakage. From the cross-sectional image of Fig. 10c, it can be confirmed that the flexibility of the fiber can be maintained because the tungsten particles are dispersed only on the surface and have negligible impact on the backside of the fabric. In addition, the thickness of the coated shielding fabric was measured to be 1.2 ± 0.02 mm.

Table 4 shows the shielding performance evaluation results for the tungsten-coated shielding fabric. The thickness of the shielding fabric was approximately 1 mm, while that of the tungsten coating was 0.2 mm. The thickness was measured by randomly selecting 10 points of the fabricated shielding fabric within 1 m^2^ area, and a uniform thickness without error was confirmed.

**Table 4:** Shielding performance evaluation result of tungsten-coated shielding fabric.

Radiation type	kVp	Mean of exposure (µR)			Shielding rate (%)	
		No shield	Shielding fabric	Standard lead	Shielding fabric	Standard lead
X-ray	40	106.90	21.27	0	90.10	100
	60	381.63	69.28	6.47	81.85	98.30
	80	799.70	172.37	48.87	78.45	93.89
	100	1318.33	338.97	144.83	74.29	89.01
	120	1648.33	458.07	207.43	72.21	87.42

In addition, the manufactured shielding fabric showed high shielding performance in the low tube-voltage range of 40–60 kVp. A shielding efficiency of 72.21% was measured even at 120 kVp, and the standard lead with the same thickness of 0.2 mm showed a shielding efficiency of 87.42%, showing a difference of approximately 15%. Although the shielding efficiency of lead is higher, the woven fabric can highly effective in practice because has fiber flexibility.

## Discussion

Most existing shielding fibers used as medical radiation shields are manufactured using a process technology of coating a nonwoven fabric with a liquid binding a shielding material mixed with a polymer material^30^. Such process technologies are difficult to mass-produce, and may have limitations in terms of product size for maintaining a coating of desired thickness^31,32^. In this study, a method of melt-spraying a single material, tungsten powder, onto the fiber, which is the base material, in a certain amount was researched and developed. To this end, a plasma thermal coating method was proposed because tungsten particles with high melting points must be provided in a semi-melted state. Various process technologies are available for shield manufacturing using tungsten, which is known to be the most effective shielding material that can replace lead^33^. However, as tungsten does not have adequate workability, it is difficult to produce a shield of the desired shape^34^. Therefore, films and sheets are mainly manufactured using a liquid form of a mixture of polymer materials and tungsten powder^35^. For the process technology to use a metallic eco-friendly shielding material such as tungsten, a forging process and a plasma thermal spray coating method for producing a thin shield may be suitable. The plasma thermal spray coating method used in this study enables mass-production of shielding clothing fabrics because it involves an automated process to keep the thickness of the shielding material constant. The method of coating the particles in the semi-molten state could allow for using mixed materials with no significant difference in the melting point in the future. The disadvantage is that the fabric to be coated must have the ability to withstand high temperatures. Nevertheless, the advantage offered by the shielding garment, i.e., flexibility (a characteristic of the fiber), is considered to outweigh this disadvantage. In addition, the proposed method is more convenient than controlling the thickness at a constant temperature and pressure, such as in a calendar process or a binder process^36^.

The radiation used in medical institutions requires the minimum energy that can penetrate the human body, and the fabric developed in this study can quantitatively control the amount of shielding material. Hence, it is possible to manufacture a thin film by applying a coating thinner than 0.2 mm. In the previous study, the shielding sheet produced by mixing tungsten and polymer material through a liquid coating process showed a shielding rate of 72% at 100 kVp for 1.5 mm thickness^37^. The 1.2 mm thick shielding fiber in this study showed a shielding performance of 72% with highly effective spraying. In this study, the shielding performance of a shielding fabric manufactured using a plasma thermal spray coating process with tungsten, a shielding material, was evaluated. However, the scope of its application is expected to be expanded if research on composite shielding materials is conducted in the future. Radiation shields used during medical procedures are primarily intended to protect patients or medical personnel, but the shield thickness should be adjusted to minimize obstruction of the medical procedures. To accomplish this, a thin, flexible shield made of a fiber material is required. Therefore, future shielding suits should be manufactured in consideration of the range of physical activity of medical staff.

## Conclusion

A shielding fabric was developed through a plasma thermal spray coating process using tungsten, a representative material that can replace lead, which is currently used for medical radiation shields. When the shielding performance of 0.2 mm thick tungsten-coated shielding fabric was compared with that of 0.2 mm standard lead, the former was approximately 15% lower. Nevertheless, since the fiber maintains its flexibility, it is considered to be suitable as a fabric for use as shielding clothing in medical institutions. Additionally, since the thickness of the tungsten coating can be controlled through the plasma thermal spray process, it is considered that it is possible to manufacture a shielding fabric that can control the shielding performance.

## Data Availability

All data generated or analyzed during this study are included in this published article.
